# Takotsubo cardiomyopathy after an upper and lower endoscopy: a case report and review of the literature

**DOI:** 10.1186/s13256-019-2014-y

**Published:** 2019-03-25

**Authors:** Ashruta Patel, Yunseok Namn, Shawn L. Shah, Ellen Scherl, David W. Wan

**Affiliations:** 1 Philadelphia College of Osteopathic Medicine – Georgia Campus, 625 Peachtree Road NW, Suwanee, GA 30024 USA; 2 Department of Medicine, Division of Gastroenterology and Hepatology, New York-Presbyterian Hospital/Weill Cornell Medicine, 1305 York Avenue, New York, NY 10022 USA

**Keywords:** Endoscopy, Takotsubo cardiomyopathy, Stress cardiomyopathy, Broken heart syndrome, Apical ballooning syndrome

## Abstract

**Background:**

Gastrointestinal endoscopies are safe and follow guidelines that emphasize patient care.

Although adverse outcomes are rare, high-risk patients may be predisposed to certain events.

**Case presentation:**

We report a unique case of a Caucasian woman with takotsubo cardiomyopathy following an upper and lower endoscopy.

**Conclusions:**

Our report suggests the importance of understanding possible endoscopic complications in patients who may experience stress cardiomyopathy.

## Background

Takotsubo cardiomyopathy (also known as stress cardiomyopathy, broken heart syndrome, or apical ballooning syndrome) is defined as a transient systolic dysfunction with diffuse wall motion abnormalities, often mimicking the presentation of an acute coronary syndrome (ACS) but without evidence of obstructive coronary artery disease (CAD) or plaque rupture [1–3]. The acute presentation, electrocardiogram (ECG) findings, and cardiac enzymes are similar to those seen in ACS [4]. It is thought that the condition largely affects postmenopausal older women exposed to intense physical or emotional stress [4]. Gastrointestinal (GI) endoscopies are done on the basis of evidence-based guidelines that prioritize safety and high-quality care [5]. To date, there have only been six reported cases of stress cardiomyopathy in patients after endoscopy. We present the second case of takotsubo cardiomyopathy following both an upper and lower endoscopy. This case is published to help other health professionals understand what medical management approaches have been used when treating at-risk patients who underwent endoscopies and experienced symptoms of stress cardiomyopathy, because this presentation is generally uncommon.

## Case presentation

A 73-year-old Caucasian woman with a past medical history (PMHx) of esophageal dysmotility, gastroesophageal reflux disease (GERD), lymphocytic colitis, chronic obstructive pulmonary disease (COPD), essential hypertension (HTN), hyperlipidemia (HLD), neuropathy, and depression presented with substernal pleuritic chest pain and lightheadedness that began 2 hours after an uncomplicated outpatient upper and lower endoscopy. She did not have any known allergies. Her family history was significant for myocardial infarction (MI) in her father and cerebrovascular accident in her mother. She was married with two children, retired, previously worked for an advertising agency, and resided in New York City. She smoked one pack of cigarettes per day for 30 years and quit in 2001. She drank two alcoholic drinks per night. Medications taken prior to admission, during hospitalization, and after discharge included a 10 mg oxybutynin extended-release oral tablet once daily for urinary symptoms, a 40 mg omeprazole oral tablet once daily for GERD, a 10 mg amlodipine oral tablet once daily for HTN, a 300 mg bupropion extended-release oral tablet once daily for depression, a 20 mg escitalopram oral tablet once daily for depression, a 100 mg topiramate oral tablet once daily for neuropathy, a 50 mg tramadol oral tablet as needed every 4 hours for pain, and a 135 mg fenofibric acid delayed-release oral tablet once daily for HLD. Prior to presentation, the patient had undergone three endoscopies, after which her postprocedure course was uncomplicated. Upper and/or lower endoscopies were done on March 11, 2013, May 29, 2014, and December 3, 2015, for epigastric abdominal pain, periumbilical abdominal pain, and chronic diarrhea, respectively.

Biopsies were taken throughout the esophagus, stomach, duodenum, jejunum, ileum, and colon. The upper endoscopy and lower endoscopy took 5 and 25 minutes to complete, respectively.

In the emergency room, her vital signs were as follows: afebrile (36.8 °C), heart rate of 101 beats/min, blood pressure of 116/59 mmHg, respiratory rate of 16 breaths/min, and oxygen saturation of 93% on room air. She was a well-appearing woman, alert, and in no acute distress. She was well hydrated and well nourished. Her skin color, texture, and turgor were all normal without any suspicious rashes or lesions. Her head was normocephalic and atraumatic without any masses, lesions, or tenderness. Her eye examination included anicteric sclera with pupils that were equally round and reactive to light and with intact extraocular movements. Her ear, nose, and throat examination were all normal. Her neck was supple without any adenopathy. Her thyroid was of normal size and symmetric without any bruits. Her lungs were clear to auscultation without any wheezing, rhonchi, or rales. Her heart sounds included a regular rhythm and rate without murmurs, rubs, or gallops. Her abdominal examination revealed a soft, nontender abdomen, normoactive bowel sounds, and was nonsignificant for masses or organomegaly. Her extremities did not show any deformities, edema, skin discoloration, clubbing, or cyanosis and had good capillary refill. No joint swelling, deformity, or tenderness was observed. Her peripheral pulses were normal. The patient was alert and oriented to person, place, and time. Her speech was fluent with appropriate repetition and comprehension. Cranial nerves II–XII were intact without any deficits. Her gait was normal and steady. Her sensation (light touch, pinprick, position sense, and vibration sense) was grossly intact. Her reflexes were 2+ and symmetric at the biceps, triceps, knees, and ankles. She had no pronator drift of outstretched arms; her muscle bulk and tone were normal; and she had full strength bilaterally.

Initial laboratory studies revealed a hemoglobin level of 11.5 g/dl (normal range for females, 12.0 to 15.0 g/dl), which was the patient’s baseline hemoglobin; a troponin I level of 8 ng/ml (normal range, 0 to 0.4 ng/ml); and a B-type natriuretic peptide level of 2900 pg/ml (normal range, up to 100 mg/L). Other laboratory findings, including electrolytes, liver function tests, renal function tests, complete blood count, serology, and urinalysis, were all within normal limits.

**Fig. 1:**
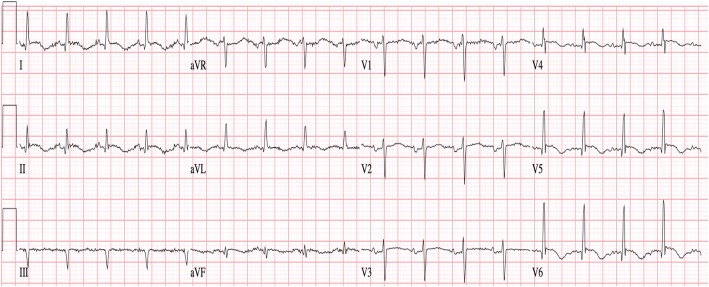
Initial electrocardiogram showing T-wave inversions in leads I, II, and V4–V6 with submillimeter ST elevations in V4–V6

**Fig. 2:**
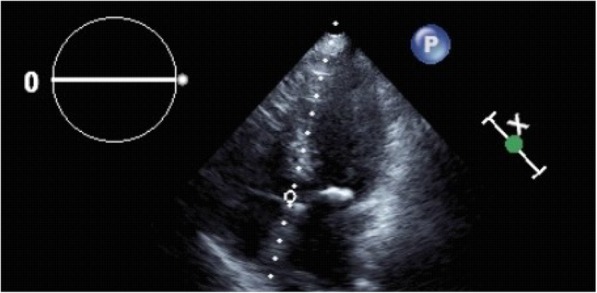
Transthoracic echocardiogram revealing apical hypokinesis

**Fig. 3:**
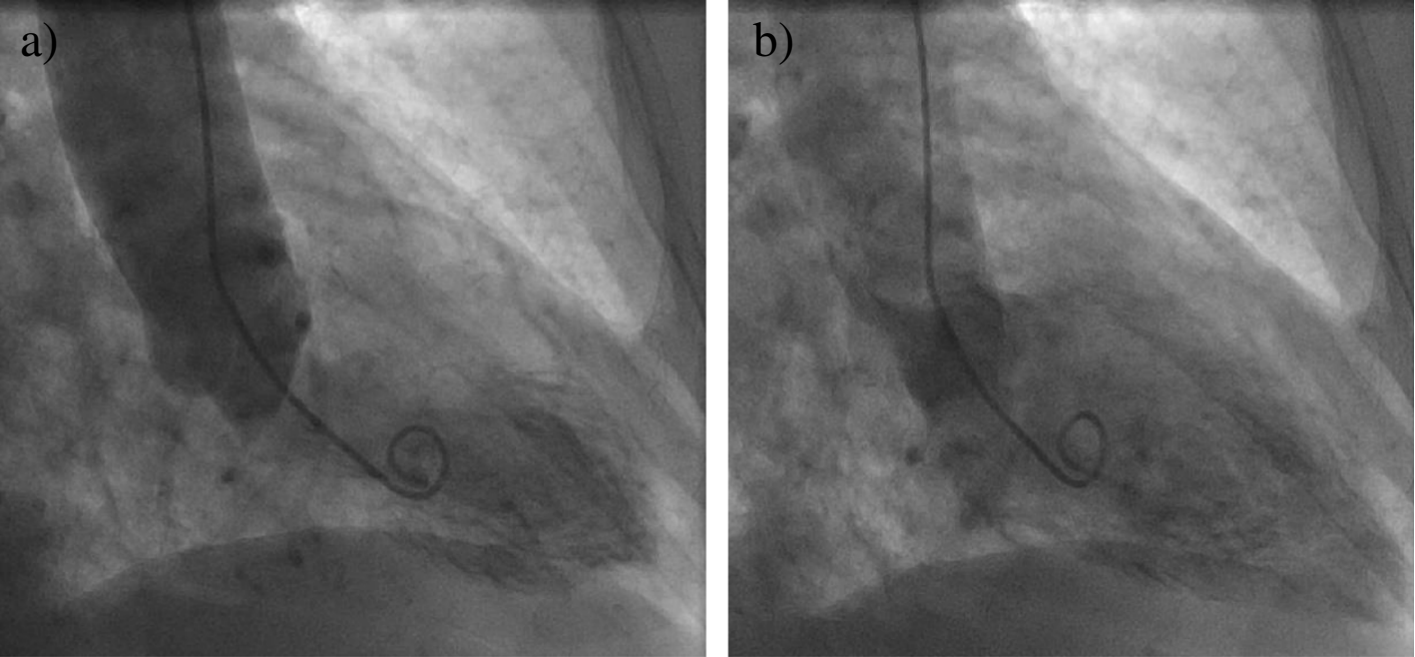
Left ventricular angiography in (**a**) diastole and (**b**) systole demonstrating severe hypokinesis of the inferolateral, anterolateral, lateral, and posterior left ventricle wall segments in setting of nonobstructive coronary artery disease

**Fig. 4:**
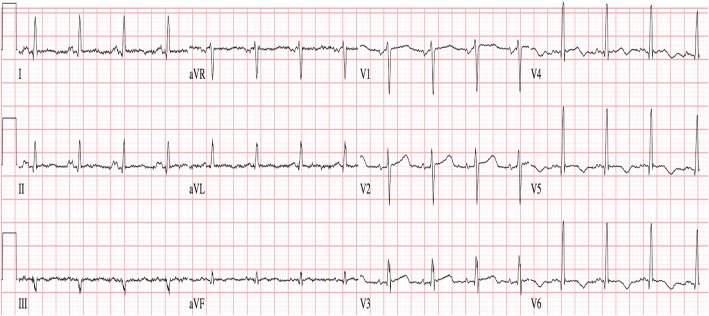
Repeat electrocardiogram obtained 24 hours after the initial electrocardiogram showing less marked T-wave inversions in V5–V6 and resolved submillimeter ST elevations

An initial ECG was notable for T-wave inversions in the anterolateral leads and submillimeter ST elevations in the V4–V6 precordial leads, concerning for ACS (Fig. 1). A bedside transthoracic echocardiogram (TTE) revealed apical hypokinesis (Fig. 2), and computed tomography of the chest, abdomen, and pelvis did not reveal pulmonary emboli or acute abdominal processes. Left heart catheterization demonstrated nonobstructive CAD with a left ventriculogram of 45% and diffuse wall hypokinesis, consistent with a diagnosis of takotsubo cardiomyopathy, thought to be precipitated by the patient’s recent upper and lower endoscopic procedures (Fig. 3a and 3b). The patient’s angina resolved after the procedure, and repeat ECG revealed less marked ST depressions and resolved ST elevations (Fig. 4). She was discharged home on hospital day 7. She did not require any further intervention or medical management.

The patient did well after discharge. After nine months post-discharge, she was admitted for worsening lower extremity edema. The TTE at the time was significant for a high left ventricular outflow tract (LVOT) gradient (peak LVOT gradients of 42 mmHg at rest and 122 mm Hg with Valsalva maneuver). Her ejection fraction (normal range, 55–70%) at the time was 81%, and pertinent results of TTE included fibrocalcific changes of the aortic valve with mildly reduced opening; moderate mitral annular calcification; systolic anterior motion of the anterior mitral valve leaflet; and normal functioning of the left atrium, right ventricle, tricuspid valve, and pulmonic valve. She was discharged with instructions on avoiding diuresis and beginning initiation of metoprolol (6.25 mg every 6 hours) for negative inotropy and to decrease systolic anterior motion.

## Discussion and conclusions

Takotsubo cardiomyopathy is a reversible cardiomyopathy that typically occurs in older women over the age of 50 and can mimic ACS [6, 7]. It can be diagnosed in several ways, including one or more of the following criteria: “transient left ventricular dysfunction presenting as apical ballooning or with focal wall motion abnormalities; an emotional, physical, or combined trigger; triggers of neurologic disorders; new ECG abnormalities; elevated cardiac biomarker (troponin and creatine kinase) levels; no evidence of infectious myocarditis; and/or postmenopausal women” [8].

Although the etiology is unknown, the vast majority of stress cardiomyopathies are thought to have an underlying emotional (for example, griefor anger) and/or physical (for example, postsurgical or malignant) trigger [7, 9]. There have been a total of six reported cases of post-GI endoscopy-induced takotsubo cardiomyopathy [7, 10–13]. Table 1 summarizes the procedures, associated cardiac findings, management, and etiologies thought to be responsible for stress cardiomyopathy in these reported cases [7, 12, 13]. Two of these studies are not included in the chart because they were published in the Japanese language [10, 11].

**Table 1:** Summary of takotsubo cardiomyopathy cases after gastrointestinal endoscopy procedures [7, 12, 13]

Authors (year published)	Patient age and comorbidities	Procedure and indication	Symptoms, postprocedure findings, follow-up, cardiac catheterization outcomes
Misbahuddin Mohammad *et al.* (2011) [12]	60-year-old postmenopausal woman with a PMHx of HTN, HLD, and Graves disease	Colonoscopy: syncope following abdominal pain, bloody diarrhea with loss of consciousness, seizure-like movements, and loss of bowel/bladder control	Symptoms: anginal chest pain Day 1 post-colonoscopy: ST segment elevation in leads V3–V5; troponin: 1.94 ng/ml; EF of 15–20%; midventricular to apical akinesis and basal left ventricular hyperkinesis 2 weeks post-colonoscopy: EF of 70–75% with no wall motion abnormality; troponin level < 0.04 mg/ml; resolution of ECG changes Cardiac catheterization: normal
Jong Won Yu *et al.* (2016) [7]	44-year-old woman with no relevant PMHx	EGD: screening	Symptoms: chest discomfort, dyspnea, hypertensive and tachycardic Day of EGD procedure: ST elevation in leads I, aVL, II, III, and aVF; ST depression in leads V4–V6; troponin: 5.39 ng/ml; hypokinetic mid-left ventricle 2 to 3 days post-EGD: normal cardiac enzymes Cardiac catheterization: normal
Jong Won Yu *et al.* (2016) [7]	45-year-old woman with a PMHx of a gastrointestinal stromal tumor (GIST)	EGD: follow-up of GIST	Symptoms: chest discomfort, hypotensive, tachycardic, and decreased oxygen saturation Day of EGD procedure: normal ECG; troponin: 3.79 ng/ml; hypokinetic mid-left ventricle; EF of 45% 2 to 7 days post-EGD: resolution of findings
Soo Ryang Kim *et al.* (2011) [13]	66-year-old woman with a PMHx of HLD	EGD and colonoscopy: screening	Symptoms: nausea, loss of consciousness, cyanosis, and respiratory arrest Day of EGD and colonoscopy: ventricular fibrillation with slight ST elevation in V2-V3; troponin ≥ 2.0 ng/ml; severely hyperkinetic base of the left ventricle 11 days post-EGD and postcolonoscopy: normal ECG and normal cardiac enzymes 2 months post-EGD and post-colonoscopy: normal ultrasound Cardiac catheterization: normal

*Abbreviations: ECG* Electrocardiogram, *EF* Ejection fraction, *EGD* Esophagogastroduodenoscopy, *HLD* Hyperlipidemia, *HTN* Essential hypertension, *PMHx* Past medical history

MI has a clinical presentation similar to that of takotsubo cardiomyopathy without coronary artery obstruction; however, there are significant differences in treatment, prognosis, and complications, possibly due to underlying emotional and/or physical stress, triggering catecholamine excess, and sympathetic nervous system hyperactivity [6, 7, 10, 14–17]. Examples of stressors include surgery, medical conditions (trauma, sepsis, stroke, malignancy, acute respiratory failure), outpatient procedures (chemotherapy, endoscopy, biopsy, stress testing), and exacerbation of COPD [16]. It is crucial to distinguish ACS from takotsubo cardiomyopathy. We report a rare case of stress cardiomyopathy after upper and lower endoscopy in a patient with no known history of cardiopulmonary disease. Though the mechanism in our patient’s case is unclear, it is thought that perhaps the insertion of the endoscopy scope triggered an overdrive of the sympathetic nervous system, resulting in tachycardia-induced cardiomyopathy [7, 9]. It is important for health professionals to be aware of potential post-endoscopic cardiac complications in those patients who may be susceptible to stress cardiomyopathy per diagnostic criteria [8].

In the United States, stress cardiomyopathy is more common in post-menopausal women and in those who have cardiovascular risk factors [18]. Studies have shown takotsubo cardiomyopathy to be associated with increased brain natriuretic peptide levels compared with ST-elevation myocardial infarction (STEMI) [19]. The product of peak troponin I levels and left ventricular ejection fraction (LVEF) have been studied and can help distinguish between takotsubo syndrome and STEMI [20]. Troponin-LVEF product was lower in takotsubo syndrome when compared with  STEMI (*p* < 0.001) [20].

There are not any reliable ECG findings in the acute phase (that is, within 12 hours of trigger or symptom(s) onset) that can differentiate stress cardiomyopathy from ACS [15, 16]. Changes during the acute phase include ST-segment elevation, new left bundle branch block, or ST-segment depression. ECG changes that develop 24–48 hours after symptom(s) or trigger may include Q waves with deep and widespread T-wave inversion with QT prolongation [15]. Takotsubo cardiomyopathy can be differentiated from acute MI by observing T waves: negative T waves in lead aVR and no negative T wave in lead V1 [21]. In addition, stress cardiomyopathy and ST-segment elevation greater than or equal to 5.5 mm are linked with increased risk of complications [22].

Management is supportive; however, risk stratification can be used when determining treatment [15]. Those with LVEF > 45% and no complications are at low risk. High-risk patients can be monitored closely in a unit with telemetry and resuscitation equipment. Sympathomimetic medications and ionotropic agents are contraindicated; however, beta-blockers can be considered in those who are at high risk with reduced LVEF. Levosimendan can be considered in those with severe cardiogenic shock and end-organ failure when mechanical support is unavailable [15]. Examples of mechanical support include intra-aortic balloon counterpulsation, extracorporeal membrane oxygenation, or temporary left ventricular assist devices. It is recommended that patients be followed for 3–6 months post-discharge [15]. Several medications can be considered for management of patients with stress cardiomyopathy. Patients with low complication risks can be considered for early discharge (that is, if LVEF is > 45%) or starting heart failure medications (if LVEF is 35–45%). Angiotensin-converting enzyme (ACE) inhibitors should be avoided in patients with a normal cardiac output because there is potential for changes in peripheral sympathetic nerve activity with low peripheral vascular resistance [15]. Those who are at high risk for complications should consider stopping sympathomimetic agents. Inotropes are generally contraindicated. High-risk patients can be started on beta-blockers when hemodynamically stable, with atrial or ventricular tachyarrhythmias, and in those with hemodynamically significant LVOT obstruction (LVOT obstruction > 40 mmHg and systolic blood pressure < 110 mmHg). Selective alpha-1 agonists are another option in those with LVOT obstruction. For anticoagulation therapy, oral anticoagulation with dual antiplatelet therapy or unfractionated/low-molecular-weight heparin can be started upon initial evaluation as clinicians may suspect MI, and once excluded, anticoagulation can be stopped. In those who have intraventricular thrombus and without high risk of bleeding, anticoagulation is recommended until left ventricular function is recovered and thrombus is resolved. Of note, beta-blockers, ACE/angiotensin receptor blockers, and aspirin may not reduce recurrence and may not have any preventative benefits for takotsubo syndrome in patients [23]. Beta-blocker use before development of takotsubo syndrome may not be able to reduce the severity of the condition [24].

A little over half of patients experience a complication from takotsubo syndrome: acute heart failure (most common), involvement of right ventricle, LVOT obstruction, mitral regurgitation, cardiogenic shock, arrhythmias, left ventricular thrombus formation, pericardial tamponade, and ventricular wall rupture [15, 16]. In-hospital mortality (1–5% of patients) is usually due to refractory cardiogenic shock or ventricular fibrillation [25]. Increased levels of brain natriuretic peptide and higher white blood cell counts upon admission have been associated with higher risk of in-hospital cardiac complications [26]. Cardiac abnormalities arising from stress cardiomyopathy are generally associated with favorable prognosis because these changes are reversible [15, 16]. Left ventricular contraction returns to normal over a couple of weeks. Therefore, clinicians should be aware of rare stress cardiomyopathy  presentation that can result after upper and lower endoscopies and use current literature to determine the optimal options for management.

## Abbreviations

ACE: Angiotensin-converting enzyme
ACS: Acute coronary syndrome
CAD: Coronary artery disease
COPD: Chronic obstructive pulmonary disease
ECG: Electrocardiogram
EGD: Esophagogastroduodenoscopy
GERD: Gastroesophageal reflux disease
GI: Gastrointestinal
GIST: Gastrointestinal stromal tumor
HLD: Hyperlipidemia
HTN: Essential hypertension
LVEF: Left ventricular ejection fraction
LVOT: Left ventricular outflow tract
MI: Myocardial infarction
PMHx: Past medical history
STEMI: ST-elevation myocardial infarction
TTE: Transthoracic echocardiogram
